# Acceptability of wearable devices for measuring mobility remotely: Observations from the Mobilise-D technical validation study

**DOI:** 10.1177/20552076221150745

**Published:** 2023-02-01

**Authors:** Alison Keogh, Lisa Alcock, Philip Brown, Ellen Buckley, Marina Brozgol, Eran Gazit, Clint Hansen, Kirsty Scott, Lars Schwickert, Clemens Becker, Jeffrey M. Hausdorff, Walter Maetzler, Lynn Rochester, Basil Sharrack, Ioannis Vogiatzis, Alison Yarnall, Claudia Mazzà, Brian Caulfield

**Affiliations:** 1 Insight Centre for Data Analytics, O’Brien Science Centre, 8797 University College Dublin, Dublin, Ireland; 2 School of Public Health, Physiotherapy and Sports Science, 8797 University College Dublin, Dublin, Ireland; 3 Translational and Clinical Research Institute, Faculty of Medical Sciences, 5994 Newcastle University, Newcastle upon Tyne, UK; 4 5983 Physiotherapy Department, The Newcastle Upon Tyne Hospitals NHS Foundation Trust, Newcastle Upon Tyne, UK; 5 INSIGNEO Institute for in silico Medicine, 7315 The University of Sheffield, Sheffield, UK; 6 Department of Mechanical Engineering, 7315 The University of Sheffield, Sheffield, UK; 7 Center for the Study of Movement, Cognition and Mobility, Neurological Institute, 26738 Tel Aviv Sourasky Medical Center, Tel Aviv, Israel; 8 Department of Neurology, 15056 University Medical Center Schleswig-Holstein Campus Kiel, Kiel, Germany; 9 Gesellschaft für Medizinische Forschung, 38697 Robert-Bosch Foundation GmbH, Stuttgart, Germany; 10 Department of Physical Therapy, Sackler Faculty of Medicine & Sagol School of Neuroscience, 26745 Tel Aviv University, Tel Aviv, Israel; 11 Department of Neuroscience and Sheffield NIHR Translational Neuroscience BRC, 7318 Sheffield Teaching Hospitals NHS Foundation Trust, Sheffield, UK; 12 Department of Sport, Exercise and Rehabilitation, 5995 Northumbria University Newcastle, Newcastle upon Tyne, UK

**Keywords:** Usability, wearable sensors, mixed methods, acceptability, digital outcomes

## Abstract

**Background:**

This study aimed to explore the acceptability of a wearable device for remotely measuring mobility in the Mobilise-D technical validation study (TVS), and to explore the acceptability of using digital tools to monitor health.

**Methods:**

Participants (*N* = 106) in the TVS wore a waist-worn device (McRoberts Dynaport MM + ) for one week. Following this, acceptability of the device was measured using two questionnaires: The Comfort Rating Scale (CRS) and a previously validated questionnaire. A subset of participants (*n* = 36) also completed semi-structured interviews to further determine device acceptability and to explore their opinions of the use of digital tools to monitor their health. Questionnaire results were analysed descriptively and interviews using a content analysis.

**Results:**

The device was considered both comfortable (median CRS (IQR; min-max) = 0.0 (0.0; 0–20) on a scale from 0–20 where lower scores signify better comfort) and acceptable (5.0 (0.5; 3.0–5.0) on a scale from 1–5 where higher scores signify better acceptability). Interviews showed it was easy to use, did not interfere with daily activities, and was comfortable. The following themes emerged from participants’ as being important to digital technology: altered expectations for themselves, the use of technology, trust, and communication with healthcare professionals.

**Conclusions:**

Digital tools may bridge existing communication gaps between patients and clinicians and participants are open to this. This work indicates that waist-worn devices are supported, but further work with patient advisors should be undertaken to understand some of the key issues highlighted. This will form part of the ongoing work of the Mobilise-D consortium.

## Introduction

Healthcare, like most industries, is undergoing a digital transformation that promises to fundamentally change how data is collected and interpreted both within clinical practice and research.^1–3^ Indeed it has been said that this transformation is a “Gutenberg moment” as digital tools offer new insights into health outside of traditional self-report measures or outcomes used in clinical environments, thus providing valuable observations into patients’ health behaviours and outcomes over prolonged periods of time.^4^ This is particularly promising for chronic conditions, where technological advancements may help to develop enhanced diagnosis, prevention of specific outcomes and optimal care, specifically, through the potential that remote monitoring of symptoms or disease progression may offer.^4^

**Figure 1.**
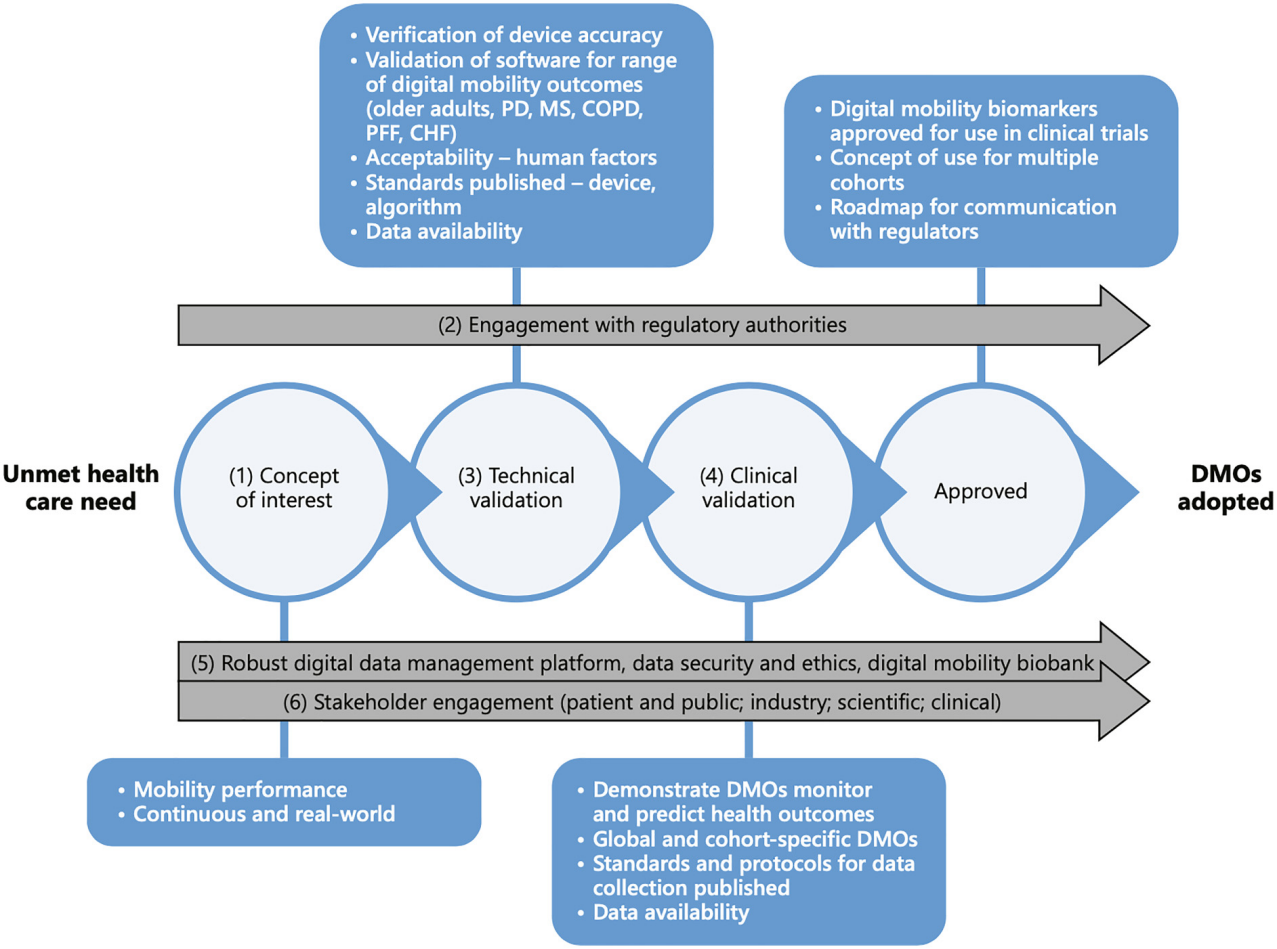
Mobilise-D project outline (as seen in Rochester *et al*., 2020).

The measurement of mobility is an area where remote, real-world, monitoring offers potential for substantial impact. Mobility is listed as the sixth vital sign^5^ and is both directly and indirectly impacted by numerous conditions while also being a critical feature of many activities of daily living.^2,6–8^ Thus, remotely monitoring this complex construct may be a valuable tool to understand the effects targeted interventions and to track overall health progress.^2^ However, despite the advances made in this area, a lack of accepted and validated tools to remotely monitor mobility remain. It is within the context of this environment that the Mobilise-D consortium was established (https://www.mobilise-d.eu). Mobilise-D is a public-private partnership funded by the European Innovative Medicines Initiative 2 Joint Under-taking, consisting of 34 partners across industry, clinical practice and academia.^2^ The overarching objective of Mobilise-D is to validate and obtain regulatory approval for digital mobility outcomes in a variety of disease states – Parkinson's disease (PD), chronic obstructive pulmonary disease (COPD), chronic heart failure (CHF), multiple sclerosis (MS) and recovery from proximal femoral fracture (PFF). The associated research programme, spanning from 2019–2024, incorporates technical and clinical validation studies of the targeted digital mobility outcome measures (Figure 1).^2^ The Technical Validation Study (TVS) ran between 2019 and 2021 adopted a multifaceted and multidisciplinary approach aiming to (i) verify the metrological performance of the included sensors, (ii) to establish the technical validity of the algorithms employed to estimate digital mobility outcomes using wearable sensor data and (iii) establish the acceptability of the deployed sensor.^9^ The Clinical Validation Study runs between 2021 and 2024 and will demonstrate that selected digital mobility outcomes quantified with the algorithms validated by Mobilise-D measure what they aim to measure, are clinically meaningful to patients and clinicians and can measure change over time.

Bodies such as the European Medicine Agency and the Food and Drug Administration have laid out the body of evidence that is required by consortiums looking to develop new digital measures and determine whether they qualify for approval.^10–13^ Within this body of evidence, there is a need to understand the patient perspective, including the acceptability of digital health tools, barriers to its implementation and their attitudes towards it.^3,14^ However, recent research has highlighted that acceptability research to date has focused on healthy adults and commonly used fitness devices, or has failed to accurately report the assessments that have taken place.^15,16^ Thus, there is a need to undertake more studies that test the acceptability of specific digital tools, and that also explore the wider concepts related to digital health across various patient populations. Within the context of the Mobilise-D TVS, participants were asked to wear the chosen wearable sensor for up to nine days, thus exploring whether it could be successfully deployed in the later Clinical Validation Study.^2^ The Mobilise-D TVS, therefore, offered an opportunity to test not only participants’ opinions concerning the device, but also a chance to explore their broader opinions around the use of digital technology in the management of their healthcare condition. Although recent research has highlighted some barriers in specific populations including COPD, CHF and PD,^17–21^ given the rapid advancements, there is a need to continue to explore this area further by understanding how multiple patient cohorts feel about the topic rather than focusing on chronic conditions in isolation.

Thus, the present study aimed to explore the acceptability of a wearable sensor to remotely monitor mobility within the Mobilise-D TVS. Additionally, we aimed to explore the acceptability of monitoring aspects of health using digital tools in the patient populations assessed as part of the Mobilise-D TVS, to help determine whether further areas of related research are needed to be undertaken as the consortium works to develop validated digital mobility outcomes for regulatory approval.

## Methods

### Study design, population and ethics

This prospective, mixed methods study took place within the context of the Mobilise-D TVS (Figure 1).^2,9^ The protocol planned to test 120 participants including healthy older adults (HA) and the five clinical cohorts included in the Mobilise-D TVS: COPD, CHF, MS, PD and PFF. This sample size was defined according to Consensus-based Standards for the selection of health Measurement Instruments guidelines for measurement properties.^12^ Full inclusion and exclusion criteria per cohort are also listed in this protocol,^9^ but included generic and condition-specific objective criteria, Montreal cognitive assessment score >15, and an ability to walk 4m independently with or without an aid.

The Mobilise-D TVS was sponsored and coordinated by The Newcastle upon Tyne Hospitals NHS Foundation Trust Participants were recruited in five sites across Europe, with the aim of recruiting up to 20 participants recruited per cohort: Tel Aviv Sourasky Medical Center, Israel; Robert Bosch Foundation for Medical Research, Germany; University of Kiel, Germany; The Newcastle upon Tyne Hospitals NHS Foundation Trust, UK; and Sheffield Teaching Hospitals NHS Foundation Trust, UK. As per the study register (ISRCTN, 12246987) data collection was due to take place across 6 months starting in April 2020. The Covid-19 pandemic delayed the start of data collection to July 2020 and its duration was extended to 12 months.

### Procedures

**Figure 2.**
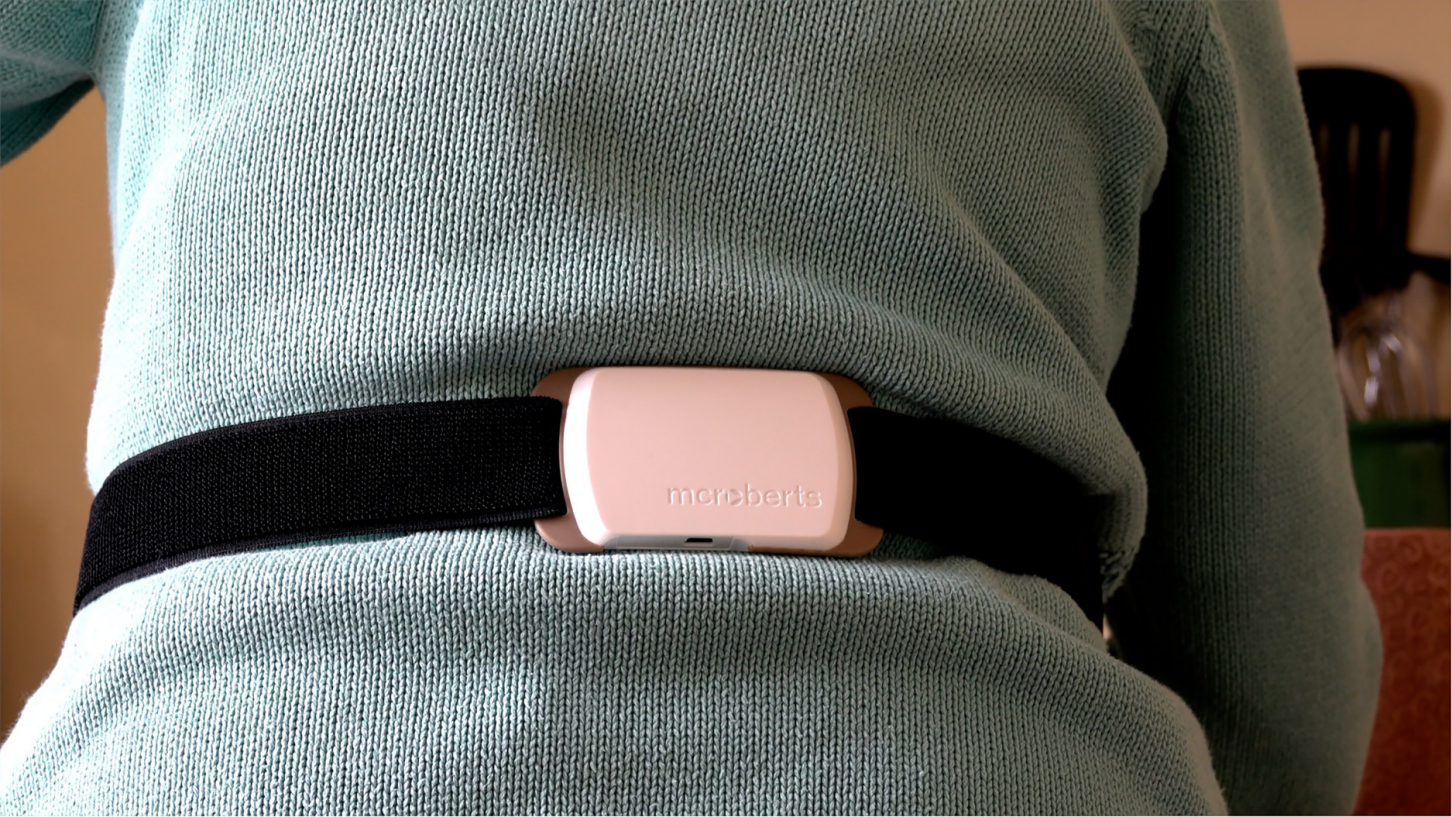
The McRoberts Dynaport MM+.

As part of the Mobilise-D TVS, participants were asked to wear the McRoberts Dynaport MM + (McRoberts B.V., Netherlands) device secured by an elasticated belt worn around their waist (106.6 × 58 × 11.5 mm) for a duration of up to nine consecutive days. During these nine days, they were instructed to complete their normal daily activities, to sleep with the device on if possible, and to only remove it to shower, swim or other related activities (Figure 2). Following the completion of this monitoring period, mixed methods were deployed to assess acceptability. Mixed methods involve purposefully collecting both quantitative and qualitative data to draw on the strengths of both methods and derive a broader perspective of the research question.^22^ Specifically, all participants completed two questionnaires to explore the comfort and acceptability of the device. Additionally, a subset of participants was asked to also complete a semi-structured interview to determine their experiences in greater depth and to explore their opinions regarding the use of technology in the management of their healthcare. The aim was to purposively recruit approximately 40% of the Mobilise-D TVS participants to complete these interviews, as this would result in a sample size of 48 interviews which is considered acceptable in most populations.^23–25^ Interviews were completed by local researchers at each site, all of whom had undergone two training and familiarisation sessions with the lead author, prior to conducting the interviews. Interviews were audio recorded and subsequently transcribed professionally verbatim before being translated, using professional services, into English, if required.

### Measures

The comfort rating scale (CRS)^26^ and the questionnaire developed by Rabinovich *et al*.,^27^ were used to measure the comfort and the acceptability of the McRoberts Dynaport MM + device respectively. The CRS is a six item 21-point Likert scale questionnaire which was developed and tested for face validity and reliability in adult populations,^26^ and has been previously deployed in older adults,^15^ children^28,29^ and people with diabetes.^30^ Participants were asked to report whether they had low agreement (i.e., ‘0’) or high agreement (i.e., ‘20’) for each of the six questions covering the topics of emotion, attachment, harm, perceived change, movement and anxiety, where low agreement signified better comfort. The Rabinovich questionnaire is split into two sections.^27^ Section A is a 12-item questionnaire on a 5-point ordinal scale. Participants were asked to note which statement reflected their experiences best, with questions covering topics such as whether the device is comfortable to wear at night, whether technical problems were experienced and how easy it was to use. Responses per question were then transformed into a numerical scale from ‘1’ to ‘5’ whereby ‘1’ signified low acceptance and ‘5’ high acceptance. Section B simply asks respondents to verbally give the device a single score between 0 and 100, and also asks them to comment on what features of the device they both liked and didn’t like. This questionnaire was developed and face validity was demonstrated through its deployment with COPD participants.^27^

The interview topic guide was also split into two components (Supplementary file 1). The first section aimed to further explore participants’ experiences of wearing the McRoberts Dynaport MM + device. Previous literature has suggested that perceived usefulness, comfort, and ease of use are critical factors of usability,^15,31–33^ thus, these were selected as the categories for which the device would be assessed. The second section was broader in nature. Participants were asked about their current healthcare and use of technology. Whether they use technology to manage their healthcare condition was explored, along with their thoughts and opinions about this domain in the future.

### Data analysis

Shapiro-Wilk tests determined data were not normally distributed (p < 0.05). Thus, non-parametric descriptive statistics were conducted for the questionnaires (median [inter-quartile range (IQR)], minimum-maximum) and reported overall and per patient cohort. A Kruskall Wallace test was used to determine differences between cohorts for each questionnaire. Interviews were analysed by AK; a post-doctoral researcher in the area of digital health with experience in qualitative methods.^1,15,34^ Interviews were analysed using a content analysis approach. The first section of the interviews was categorized deductively based on the previously listed sections of comfort, ease of use, and perceived usefulness, in line with the Technology Acceptance Model.^35^ Perceived usefulness was defined as ‘the degree to which a person believes that using a device would enhance their health’, perceived use was defined as ‘the degree to which a person believes that using a device would be free of effort’,^35^ while comfort was ‘a state of physical ease and freedom from pain or constraint’. The second section of the interviews was analysed inductively to explore participants’ current healthcare experiences and their use of and opinions towards technology in the management of their health and condition. Codes that related to these concepts were identified within the text and grouped together into meaningful themes. Specific quotations, which were deemed to represent the most important aspects of participants’ experiences were selected for inclusion by AK.

Given the primary aim of this manuscript, data saturation was considered in relation to the acceptability of the device. The second part of the interviews was considered exploratory in nature, and given the purposive method of sampling and the broad, complex topic under consideration, we cannot be certain that saturation was reached for this component across all cohorts*.* Thus, although the sample size used in this study falls within the range that saturation is typically found in,^36^ the themes related to the second part of the interviews should be interpreted with caution. Nonetheless, saturation was reached for the acceptability component of the interviews as no new information was seen in more than three interviews across the cohorts for each of the explored themes.

## Results

The study protocol aimed to recruit 120 participants, of which 111 were recruited up to January 2022. There was a shortfall in the recruitment of CHF patients due to delays and concerns associated with the covid-19 pandemic. Of the 111 participants recruited to the TVS study 106 (95.5%) completed these questionnaires. Participant demographics are listed in Table 1.

**Table 1.** Participant demographics.

	Total	CHF	COPD	HA	MS	PD	PFF
**Recruited**	106	10	17	20	20	20	19
**Country (*n* = ; %)**							
Germany	48	10	0	3	5	11	19
	(45.3%)	(100%)	(0%)	(15%)	(25%)	(55%)	(100%)
Israel	14	0	0	7	4	3	0
	(13.2%)	(0%)	(0%)	(35%)	(20%)	(15%)	(0%)
UK	44	0	17	10	11	6	0
	(41.5%)	(0%)	(100%)	(50%)	(55%)	(30%)	(0%)
**Sex (*n* = ; %)**							
Male	63	7	9	12	11	16	8
	(59.4%)	(70%)	(52.9%)	(60%)	(55%)	(80%)	(42.1%)
Female	44	3	8	8	9	4	11
	(41.5%)	(30%)	(47.1%)	(40%)	(45%)	(20%)	(57.9%)
**Age (mean; sd)**	67.6	66.8	69.4	70	48.7	69.9	80.3
	(13.4)	(11.4)	(9.1)	(9.6)	(9.7)	(7.2)	(8.4)
**Residence (*n* = ; %)**							
Community	101	9	17	20	20	19	16
	(95.3%)	(90%)	(100%)	(100%)	(100%)	(95%)	(84.2%)
Nursing home	5	1	0	0	0	1	3
	(4.7%)	(10%)	(0%)	(0%)	(0%)	(5%)	(15.8%)
**Education (*n* = ; %)**							
12 years or less	48	3	11	9	5	10	10
	(45.3%)	(30%)	(64.7%)	(45%)	(25%)	(50%)	(52.6%)
More than 12 years	58	7	6	11	15	10	9
	(54.7%)	(70%)	(35.3%)	(55%)	(75%)	(50%)	(47.4%)

CHF = chronic heart failure; COPD = chronic obstructive pulmonary disease; HA = healthy adult; MS = multiple sclerosis; PD = Parkinson's disease; PFF = proximal femoral fracture.

### Questionnaire data

The median (IQR; minimum-maximum) results from the CRS was 0.0 out of 20 (0.0; 0–20), indicative of a more comfortable device. Results were consistent across all cohorts (*p* > 0.05; Table 2).

**Table 2.** Comfort rating scale results for the McRoberts Dynaport across each cohort.

Cohort	CRS score [range = 0–20]* (median [IQR]; min-max)
Overall (*n* = 101)	0.0 (0.0; 0–20)
CHF (*n* = 9)	0.0 (0.0; 0.0–0.0)
COPD (*n* = 15)	0.0 (0.4; 0.0 −0.5)
HA (*n* = 20)	0.0 (1.0; 0.0–8.5)
MS (*n* = 20)	0.0 (1.0; 0.0–14.0)
PD (*n* = 18)	0.0 (2.3; 0.0–20)
PFF (*n* = 18)	0.0 (0.0; 0.0–2.0)

*A low score indicates high levels of comfort; CHF = chronic heart failure; COPD = chronic obstructive pulmonary disease; HA = healthy adult; MS = multiple sclerosis; PD = Parkinson's disease; PFF = proximal femoral fracture.

With regards to the Rabinovich questionnaire,^27^ results of Section A indicate high acceptability with a median score of 5.0 out of 5 (0.5; 3.0–5.0). This was further supported by Section B with a median result of 98.0 out of 100 (10.0; 50–100). Results were consistent across all cohorts (*p* > 0.05; Table 3).

**Table 3.** Rabinovich^22^ questionnaire results for the McRoberts Dynaport across each cohort.

Cohort	Section A score* [range = 1–5] (median [IQR]; min-max)	Section B score* [range = 0–100] (median [IQR]; min-max)
Overall (*n* = 100)	5.0 (0.5; 3.0–5.0)	98.0 (10.0; 50–100)
CHF (*n* = 9)	5.0 (0.3; 4.0–5.0)	97.5 (20.0; 80–100)
COPD (*n* = 15)	5.0 (0.0; 5.0–5.0)	100 (4.0; 95–100)
HA (*n* = 20)	5.0 (0.5; 3.0–5.0)	95.0 (9.0; 50–100)
MS (*n* = 20)	5.0 (0.5; 4.0–5.0)	99.0 (15.0; 70–100)
PD (*n* = 17)	5.0 (0.8; 4.0–5.0)	95.0 (13.0; 60–100)
PFF (*n* = 18)	5.0 (0.5; 4.0–5.0)	100 (6.0; 50–100)

*A high score indicates high levels of acceptability; CHF = chronic heart failure; COPD = chronic obstructive pulmonary disease; HA = healthy adult; MS = multiple sclerosis; PD = Parkinson's disease; PFF = proximal femoral fracture.

### Interviews

A total of 36 participants were interviewed from the TVS (33.6%; Table 4). Due to the problems associated with recruiting CHF participants within the Covid-19 pandemic, no CHF participants took part in these interviews. Interviews conducted as part of the TVS were split into two components. Supporting quotations are provided in Table 5.

**Table 4.** Demographics of participants that completed interviews conducted as part of the TVS.

Cohort	Total (%)
CHF	0 (0%)
COPD	6 (16.7%)
HA	8 (23.5%)
MS	12 (38.2%)
PD	5 (14.7%)
PFF	5 (14.7%)
**Country**	
Germany	8 (22.2%)
Israel	11 (30.6%)
UK	17 (47.2%)
**Gender**	
Male	20 (55.6%)
Female	16 (44.4%)
**Age**	66.7 (14.8)
**Residence**	
Community	36 (100%)
**Education**	
12 years or more	26 (72.2%)
Less than 12 years	10 (27.8%)

CHF = chronic heart failure; COPD = chronic obstructive pulmonary disease; HA = healthy adult; MS = multiple sclerosis; PD = Parkinson's disease; PFF = proximal femoral fracture.

**Table 5.** Quotations related to emerging themes.

Theme	Quotation
**McRoberts device**	
Ease of use	*“It didn’t disturb me while walking. I slept with it without any problem. It was easy. It wasn’t difficult.” –* PD, Israel, Male
	*“Yes, at first I had to get used to the device. But when I found out that it wasn't witchcraft, it became routine and I took it off in the evening and put it back on in the morning.” –* PFF, Germany, Male
	*“I never had any problems with it*” – COPD, UK, Female
	*“At first I was quite conscious of it. And when I say at first, I’m talking about the first few hours, really. But after that, it just became a thing to do, like cleaning your teeth, really. It didn’t interrupt with what I was doing.” –* MS, UK, Male
Perceived usefulness	*“It doesn’t bother me because whatever that did, it's on there for your purpose, not for mine.” –* MS, UK, Male
Comfort	*“Because I didn't want it next to my skin and I can find it quite difficult to get off to sleep. And I knew that that would be more difficult. And sleep problems with fatigue - they’re not happy partners. So, I try and get the best sleep that I possibly can and I didn’t want to jeopardize that.” –* MS, UK, Male
	*“Very comfortable. The truth is that in the beginning, on the first day of use, I put it too high. So I learned where to place the belt on the front. When I placed the belt on my belly, it rose up to my chest So I learned that I just have to place it lower on the belly, and it would remain there.” –* PD, Israel, Male
	*“If you don’t get the two pieces exactly positioned and you’ve got a bit of Velcro against your skin and I, being old, the older you get, the thinner your skin gets and you get much more sensitive.” –* COPD, UK, Female
	*“It is very comfortable. I haven’t felt any discomfort. Sometimes when I put it on the body it causes itching. For a person who has to wear it constantly, it is better to make it narrower and more compact. Then it will not be seen under the clothes.” –* HA, Israel, Female
Likelihood to wear	*“Once it was on, you very quickly get used to it being there. So, whether it's there a week or a month or, you know – a day probably doesn’t give you enough information. So, I would have thought a week is okay.” –* MS, UK, Female
	*“I knew I was wearing it for a good purpose and I think that, psychologically, you just think – well you just - you know, it's something you just - you don’t allow it to upset you because you’ve agreed to do it, you know, I agreed to do it.” -* COPD, UK, Female
	*“I wouldn’t want to wear it constantly, but if there was a period of time, you know, say you’ve got a consultation in two weeks’ time “Could you wear this for a couple of weeks and then we can assess it?” I’d be fine with something like that. I just feel, you know – it's strange, cause this, which is for trial purposes, then I’m like “Yeah, great” but if it was like “You have to wear this for the rest of your life” I’d be like “Okay” [sounds sad]. Because I feel like I’m helping, it's a lot easier for me to take than if I had to wear it for the rest of my life, type thing.” -* UK, MS, Male
**Use of technology in healthcare and the management of their condition**	
Communication with healthcare professional	*“At that point I had no idea that it might be MS. I think they initially very quickly got me to the hospital because they were perhaps more concerned that it could be a cancer. And so, the day that I went to the GP I was straight off to the hospital. I was in neurology by the end of the day. And they did MRI scans and a few other tests, I think, for worrying it could be cancer. Ruled that out. But at that point then they said “Well, could be MS here” and that was a fairly difficult one to adjust to. It is a difficult one, because I’ve got no family history of it, I’m a man, which is more unusual for MS. So, you start to question where does it come from? Why has it happened? What's triggered it? Is it something I’ve done? Could I have prevented it? So, that - you start to doubt and question yourself a little bit. Yeah, wonder what does it mean for the rest of your life? What's the future hold?” –* MS, UK, Male
	*“Yes, I suppose so. Can I just tell you that twice, I’ve been on these rehab courses. I find that when I’m in that setting, I actually do the course but when I'm at home, it just fades away. I don’t do anything. I don’t know if it's the group setting or what it is but they say, “You can do these exercises at home.” But I don’t. It just falls flat for me.” –* COPD, UK, Male
	*“I suppose that's what I find frustrating about MS is that we tend to see the symptoms and we make our diagnoses based on that. What we don’t know is what the future holds. Where the disease is progressing. And how we can stop it progressing. You know. We take a tablet because we think it will help us not relapse, but we don’t know where the indications are.” –* MS, UK, Female
Altered expectations	*“And it was pretty busy at work with all that and then I got my diagnosis. Immediately, I wasn’t allowed to do all of this stuff that I wanted to do and was able to do previously. And I was put into a restricted role, sort of an office job, with bizarrely more scrutiny than I had when I was on the road. And I found it exceptionally difficult. In fact it was very stressful, actually. And it came to a point where I was gonna ask to leave and but it was offered to me, would I like to go on medical retirement.” –* MS, UK, Male
	*“Well, over the years, my mobility is the, kind of, thing that's got worse. I'm not as mobile as I used to be. I'm breathless and I get breathless very easily but I'm also constantly tired, like, you know, I’m always tired, which I don't like, but that's the way I’ve become, so I just want to sleep all the time.” –* COPD, UK, Male
	*“The fact that my mobility is really hard now. Really difficult. And, you know, just sort of getting a cup of coffee sometimes, I think “Do I want one? Do I really need one?” … It's one of those things that never goes away, you’re always – well, from my point of view, anyway – you sit there and you’re thinking “Is tomorrow the last time I’ll be able to stand up?”, or, you know. You start to think of things like that.” –* MS, UK, Male
The use of technology	*“I have to see a purpose in things and I perceive them as being a kind of watering down of stuff which we should be doing normally. You know, I don’t need to tell something to put the kettle on for me, I can do it myself.” –* MS, UK, Male
	*“Oh, well, I’d like there to be a diagnostic machine that I could stick my finger in and it would just go, right, this is you know, like a car? I like the idea of being able to be diagnosed without running through, sort of, things that could be – you know, they – you know, they picked up my osteoporosis rather late in the day.” –* COPD, UK, Female
	*“Well, yes, particularly for yourselves. The problem is, with all medical experiments, it's long term. There's no quick fix for anything. Possibly, what you're doing now won't be of any benefit to me, but it might be beneficial for somebody in, say, two or three years' time, against Parkinson's or who has a problem with the walking aspect.” –* PD, UK, Male
Trust	*“You’re in a position of trust with the clinician anyway, so you’re relying on an expert to support you in whatever issues you’re experiencing and if they say “Look, I’ve got this technology. It will tell me this. It will help me provide you with an appropriate care” then yes. I think it's a good thing”. – MS, UK, Female*
	*“Well, I hope it's being handled as if it was – you know, as if it was someone's child or something you know, the utmost has gone to keep that information within the realms of medical science, as it were, and not to get out to other people. Because I think the last thing I would want to do is if I were sitting at my work and all of a sudden something popped up…. MS reacts different people- that technology is so- if I were to give all my details and all of a sudden I start finding that I’m getting, you know, advertisements through for various MS treatments or whatever, and I know that I haven’t done it, I’d upset that – I’d feel that the information that I gave the hospital or doctors or things like that had been infiltrated and passed on back to myself.” –* MS, UK, Male
	*“No. If it is between me and the doctor only, it's OK. IF the data is transferred to [company name], it's another story. If it is between me and my family doctor or orthopedist only, it's normal.” –* HA, Israel, Female
	*“No. This is not a problem. I have nothing to hide. If it can help, I’d do everything.” –* MS, Israel, Female

#### Mcroberts dynaport

##### Ease of use

The McRoberts Dynaport MM + was considered easy to interact with, primarily because little to no interaction was required with it. Indeed, the only interaction that was required was donning and doffing the device and adjusting its position during the day. The lack of direct participant feedback from the device to the participant, its position on the body, and the week-long battery life, combined to preclude any other engagement with it. Thus, once participants were confident with positioning on the body (some used the imprinted writing on the side to guide them) and how to put it on, they were confident with it for the week. Furthermore, while wearing it, all participants agreed that it did not interfere with their daily tasks or activities. The only time of day where some interference was noted was at night. Most participants who slept on their side did not notice it while sleeping. However, light sleepers, and people who slept on their back did remark that it was noticeable but continued to sleep with it for the week. It should also be noted that none of the interviewed participants had issues with dressing independently or incontinence, thus these results can only be applied to those who are fully independent.

##### Perceived usefulness

The device was not considered useful due to the lack of interaction and patient-facing feedback derived from it. It was simply noted that participants trusted it was doing ‘it's job’. That is, it was working and silently collecting the data needed. A number of participants with MS, in particular, noted that it would be nice to know if it was working, through even simply a light that glows when working. Ultimately, however, they trusted that the device, and the data acquired, would be useful for someone, be it researchers or clinicians, but that for patients, the use was likely to be more indirect in nature.

##### Comfort of the device

Comfort was at the forefront of the participants’ minds as, due to the lack of required engagement, it was the most prominent aspect for them to consider. Differences in comfort were noted across all cohorts, suggesting that this is a concept related to personal preference, rather than any specific issues linked to symptoms, conditions or the device itself.

“It's like all these things: once you've had them on for a while, the first half an hour or hour or so, you're aware of it, but after that, you just forget it's there.” – PD, UK, Male

Overall, participants remarked that the device was “forgettable” (i.e., out of sight, out of mind), however, there were some small issues linked to how comfortable it was as a result of both its size and its Velcro strap. For some, the strap was irritating to their skin unless they wore the device over clothes or ensured that the strap was overlapped onto itself appropriately. In relation to the size of the device, participants agreed that it was quite large and were initially apprehensive about it. However, it was surprising to them how comfortable it was.

##### Likelihood to wear the device

Participants were asked how long they would be willing to wear such a device. There was a mix of responses ranging from no more than a week, to a few weeks at a time. This generally coincided with how comfortable they found it, and how willing a person was to wear a device for which they received no direct benefit. Participants agreed that wearing the device for the week was reasonable in the context of a research study because it was for the benefit of research and may help others. Ultimately though, because of the lack of direct benefit to them, participants felt that wearing it for much longer than a week would become annoying and they were glad when the week was over.

“I don’t know. I wouldn’t like it on an exposed part of the body, for example, in summer. I wouldn’t like it. It may be more onerous in summer. I like wearing dresses. This is less comfortable, since the device has to be attached right on the body.” – HA, Israel, Female

#### The use of technology in healthcare and the management of their condition

Participants were asked about the management of their condition or health overall and their current or potential use of technology within that (Table 5). Experiences between participants were varied and reflected not only the specific condition, but factors such as their age and the health system of the country they reside in. Furthermore, some participants were more willing to speak about their experiences than others. Thus, the results of this section should be interpreted cautiously, while the themes outlined within it require further investigation. Indeed, some themes that were present do not directly relate to the use of technology within clinical trials, but highlight some unmet clinical needs where technology or remote monitoring may support condition management in the future. Specifically, the following themes were noted: (1) altered expectations for themselves, (2) the use of technology, (3) trust, and (4) communication with healthcare professionals.

##### Altered expectations for themselves

One of the struggles that participants commonly noted was the change from their lives and tasks prior to their diagnosis to where they are now (Table 5). For many, their condition came as a shock and was difficult to come to terms with. This resulted in denial, frustration, fear, and anxiety. Essentially they had to adjust their expectations as to what they could now do safely when compared to before. Participants had concerns regarding what the future held for them, especially as it was unclear how their conditions may progress and when. Some had already been forced to give up work or leisure activities while others remarked that they could not plan ahead to the same extent because of the unpredictable nature of their condition. This led them to change their lifestyle and behaviour in response to their symptoms, to keep them safe, and to ensure that they could maintain as much independence as possible.

“It is very inhibitory, if that's a word, of the way I would – of the way I was. So, I find it mentally… mentally, I’m finding it very difficult to adjust Because this, it has increased over the last three years. It, sort of, started off as a minor thing and, you know, but perceptible, obviously, otherwise I wouldn’t do anything.” – COPD, UK, Female

##### The use of technology

Participants were all using technology in their daily lives through mobile phones, computers, etc., for the purpose of everyday use. No participants used technology to support them in monitoring their condition or for any other healthcare purpose. A small number (*n* = 4; 11.1%) were wearing devices such as Fitbit to monitor their steps. However, they did not see this specifically as managing their condition, but rather used it out of interest or to promote physical activity in general. Furthermore, they were not clear how this type of information would help their doctors either. Thus, when questioned about how technology may help them in the future, their responses were mostly hypothetical. Participants didn’t fully understand exactly what technology could detect and what this meant for them. Some questioned whether it would know where they were, others stated they wondered ‘what the sensor is seeing’. They believe that technology has the potential to help their condition, although they are not sure how and what this may look like (Table 5). They remarked that they would like technology to provide them with information that they don’t already know such as triggering an early change in treatment and helping diagnose conditions early. Participants were therefore generally happy to be monitored for the purpose of research, but they noted discomfort at the prospect of doing this long-term.

“If there was a reason to monitor in order to bring in interventions to prevent disability, that could be a good reason for somebody. If it meant for me that there was a point where there was a procedure or something that needed to be done to prevent disability down the line then I’d quite happily wear it. But if I got that notification for the treatment to happen at the right moment rather than needing to be caught at the right moment, then that sort of thing would be a brilliant reason.” – MS, UK, Male

##### Trust

Those who noted their potential discomfort at being monitored for longer periods of time, inferred that this was the result of a lack of trust (Table 5). Although none of them explicitly mentioned that they had experienced a breach in trust, they were nonetheless aware that this could happen. This was especially true for companies or external bodies. Indeed, an unwavering trust in medics and universities was reported whereby participants were of the opinion that if these people were content that data needed to be collected and that devices were safe, then they would abide by this advice. Participants were less concerned about providing their data, or of the concept of ‘Big Brother’ watching them, as they typically felt they had nothing to hide and that the benefits of technology in healthcare outweigh the positives.

##### Communication with healthcare professionals

There was an inferred suggestion that care is generally reactive in nature, regardless of the condition they have. Participants learn about their diagnosis from their healthcare professionals, and rely on them to provide them with information about the condition, what they can do to support and manage it, how they can expect it to progress, and what is considered normal. Although participants were appreciative of the efforts of their healthcare team, and did not explicitly criticise them, they nonetheless inferred and suggested that they had unmet needs when it came to how their condition is managed (Table 5). For example, participants remarked that healthcare professionals do not fully understand the impact of their condition on their individual lives, while there is a lack of communication between general practitioners and hospital-based clinicians. There was a feeling by some that this may impact their care, or at the least, that it made them uncertain or less confident about its continuity.

## Discussion

The results of this study demonstrated that a single waist worn device (the McRoberts Dynaport MM + ) is considered acceptable for participants to monitor their mobility for week-long periods, as was the case in our study context. This study achieved its aims using mixed methods, and by recruiting a wide range of participants with a range of clinical conditions, thus making it one of the most comprehensive assessments of acceptability to date. Specifically, the device was shown to be comfortable despite its size, and didn’t interfere with daily activities, although participants did note some reluctance to wear it for longer periods of time (greater than a few weeks). Interviews also demonstrated that while there is openness to using digital tools in the monitoring of their health condition, that participants are generally not aware of what this may look like in practice or how it may benefit them. Furthermore, any monitoring must be done by institutions who they trust to manage their data, rather than for profit from companies who may use it for advertising and/or marketing. Thus, this rapidly progressing area needs to ensure that advancements are trialled with patient groups in the future to ensure acceptability continues to exist, to understand the possible barriers to implementation of any future tools that are developed, and to protect and highlight patient needs and experiences.

Assessing the usability and acceptability of wearable devices prior to their use in larger scale trials is critical to ensure that they will be worn as intended so as to prevent data loss. Despite this, researchers acknowledge that they rarely report any pilot trials that they complete,^1^ and indeed, this may explain why limited published data relating to the acceptability McRoberts Dynaport MM + device, and other similar devices, exists. A previous study in COPD participants compared multiple devices, with good acceptability noted.^27^ This study goes further by testing the device in multiple cohorts, thus strengthening the case that using waist-worn devices for remote monitoring purposes is acceptable. Furthermore, it has been previously shown that a trade-off between comfort and functionality appears to exist,^37,38^ therefore given the lack of required interaction with the device, it was important to not rely solely on one questionnaire and to specifically measure participants perceptions of the comfort of the device using the CRS. As shown, differences between cohorts regarding the comfort of the device were minimal with all of them demonstrating a low level of discomfort on a 20-point scale. Interestingly, adults have previously rated commercial wearable devices as having low acceptability,^39^ however the purpose of those devices was different and they required greater levels of interaction which may explain the high levels of acceptance in this study.

An additional strength of this study was the use of mixed methods to comprehensively explore the topic of acceptability with participants. We have previously highlighted the need to combine validated questionnaires with qualitative methods^16^ and the results of the interviews have highlighted why. Firstly, the interviews broadly supported the results of the questionnaires, thus evidencing participants’ confidence in the use of the device. However, they also highlighted that participants would only be willing to wear the device for relatively short periods of time (i.e., 1–2 weeks, possibly a month). This is possibly related to the lack of direct benefit to participants, even though the device is made to monitor movement and not motivate people to move or change their behaviour. This distinction in desired outcomes is important for participants to understand, therefore future work should explain in greater detail why direct feedback may not be provided. Additionally, as remote monitoring becomes more common, the usefulness of its outcomes may also become clearer to patients, thus potentially offering greater scope in terms of wear-time. Furthermore, although many participants in this study wore it at night, it was nonetheless noticeable which may have impacted their opinions regarding its future use. Sleep was not a target behaviour monitored in this study, therefore for future, similar studies, it is worth considering asking participants to remove the device at night to increase compliance. While this doesn’t directly influence the Mobilise-D clinical validation study, as the device will only be used for seven days at a time, it does suggest that its comfort is dependent on the person knowing that they only need to wear it for a short period of time. After this, it may become burdensome due to it being potentially more visible under lighter clothes or on outer layers in summer months, reduced motivation or because participants have no direct benefit to be gained from it.^15,38,40–42^ Thus, additional strategies would be needed for studies which plan to use devices such as the McRoberts Dynaport MM + for more than one week, or alternative devices may need to be explored. For example, other waist worn devices may be placed directly on the skin (e.g., Axivity x3 [Axivity Ltd, UK]), thus may be less visible or intrusive at night. While wrist worn devices are typically well accepted by participants but may be less accurate in terms of gait monitoring.^15,33,43,44^

With regards to the second component of the interviews, results need to be considered in line with the limitations of the study. Firstly, interviews were analysed by a single researcher. Next, participants were all using smartphone devices and therefore were likely to be representative of a group who is open to technology. Furthermore, although close to 40% of the overall TVS participants completed interviews, there is a slight bias towards those based in the UK and those with MS. The Mobilise-D TVS ran during the Covid-19 pandemic which posed a challenge across all sites due to the restrictions imposed by local research departments/ healthcare settings. Participants with COPD and CHF took longer to recruit, possibly due to the advice given to these cohorts around shielding, while local restrictions in the UK allowed testing to take place earlier than other sites. In addition, as there was a period of time between when interviews were conducted and when they were subsequently translated and uploaded for analysis, it was not fully clear how many participants were recruited for interview until quotas began to be reached in some sites. Although data saturation was reached for the primary aim of the manuscript, the broad nature of the topic in the second component of the interview, within the context of our purposive sampling, means that we cannot be certain that saturation was reach for this section. Prior to conducting the interviews, not all researchers were experienced in qualitative research methods. Next, while all researchers undertook training and follow up calls as part of the protocol, the exploratory nature of the second section of the interview topic guide may have benefited from a greater level of interview expertise to ensure sufficient depth of detail was captured. This was especially visible in the PFF cohort who's interviews focused only on the use of the McRoberts device and not on the second interview component. Finally, we did not measure the health or digital literacy of our participants, thus cannot account for the impact of this on these results.

Nonetheless, despite these limitations, the information derived from the interviews is supported by previous research,^19–23^ suggesting that there are issues related to how researchers and clinicians communicate with patients in terms of their condition, use of technology and the unmet needs that may be met with greater technological advancements. Specifically, participants noted that although they understood their diagnosis, they didn’t necessarily understand how their condition may develop or what they may expect. Such a lack of clarity has been noted before in both MS and COPD populations^36,37^ and is further emphasised by a perception that their care is either reactive in nature or that they were somewhat left alone to navigate their care between consultant visits.^20,22^ While participants did not explicitly note disappointment with their care, their experiences suggest that there is a lack of consistency in communication and that many are dealing with healthcare systems that are exceptionally busy which limits the opportunity for individualised care. Because of this, participants were open to the idea of using technology to help communicate with healthcare professionals and to support them in the monitoring and insights into their condition. This has been previously noted,^19–23^ however it is also noted that, for now, much of this is theoretical in nature. Participants are aware of the need to protect their health data and so while they trust certain institutions to maintain their data securely, they also noted that they would be disappointed, if not angry, were that trust to be breached. Thus, while the potential for digital tools and technologies is almost limitless in their minds, in reality, future innovations need to engage with patients in the development of their tools in order to make sure that they address both current challenges and future concerns.

## Conclusion

There is an opportunity for digital tools to bridge existing communication gaps between patients and clinicians through the use of individualised, objective data. Results of this study show that patients are clearly open to it and they are, in theory at least, accepting of new digital tools to help them manage their condition. However, given the rapid advancements in this area and the lack of clarity regarding what this means for them and how their data will be managed, these innovations will require additional measures/support to be put in place to make communication more transparent and suitable to patient needs. Without it, patient needs will continue to not be fully met. This study, therefore, suggests that there is a need for further research in this area, using multiple cohorts, to understand current barriers in greater depth and to consider how best to overcome them, using patient insight to guide us. Specifically for the Mobilise-D study, the use of a waist-worn device is supported in the clinical validation study as a result of this work, but further work with patient advisors will be undertaken to understand some of the key issues highlighted by participants in the Mobilise-D TVS.

## Supplemental Material

sj-docx-1-dhj-10.1177_20552076221150745 - Supplemental material for Acceptability of wearable devices for measuring mobility remotely: Observations from the Mobilise-D technical validation study Click here for additional data file. Supplemental material, sj-docx-1-dhj-10.1177_20552076221150745 for Acceptability of wearable devices for measuring mobility remotely: Observations from the Mobilise-D technical validation study by Alison Keogh, Lisa Alcock, Philip Brown, Ellen Buckley, Marina Brozgol, Eran Gazit, Clint Hansen, Kirsty Scott, Lars Schwickert, Clemens Becker, Jeffrey M. Hausdorff, Walter Maetzler, Lynn Rochester, Basil Sharrack, Ioannis Vogiatzis, Alison Yarnall, Claudia Mazzà and Brian Caulfield in Digital Health

## References

[1] Keogh A Taraldsen K Caulfield B, et al. It's not about the capture, it's about what we can learn": a qualitative study of experts’ opinions and experiences regarding the use of wearable sensors to measure gait and physical activity. J Neuroeng Rehabil 2021; 18: 78. 33975600 10.1186/s12984-021-00874-8 PMC8111746

[2] Rochester L Mazza C Mueller A, et al. A roadmap to inform development, validation and approval of digital mobility outcomes: the Mobilise-D approach. Digit Biomark 2020; 4: 13–27. 10.1159/000512513 PMC7768123 33442578

[3] Jandoo T. WHO Guidance for digital health: what it means for researchers. Digit Health 2020; 6: 2055207619898984. 31949918 10.1177/2055207619898984 PMC6952850

[4] Gopal G Suter-Crazzolara C Toldo L, et al. Digital transformation in healthcare - architectures of present and future information technologies. Clin Chem Lab Med 2019; 57: 328–335. 30530878 10.1515/cclm-2018-0658

[5] Brabrand M Kellett J Opio M, et al. Should impaired mobility on presentation be a vital sign? Acta Anaesthesiol Scand 2018; 62: 945–952. 29512139 10.1111/aas.13098

[6] Mollenkopf H Hieber A Wahl H-W. Continuity and change in older adults’ perceptions of out-of-home mobility over ten years: a qualitative–quantitative approach. Ageing Soc 2011; 31: 782–802.

[7] Leung KM Ou KL Chung PK, et al. Older Adults’ Perceptions toward Walking: A Qualitative Study Using a Social-Ecological Model. Int J Environ Res Public Health 2021; 18: 7686. 34300136 10.3390/ijerph18147686 PMC8303868

[8] Webber SC Porter MM Menec VH. Mobility in older adults: a comprehensive framework. Gerontologist 2010; 50: 443–450. 20145017 10.1093/geront/gnq013

[9] Mazza C Alcock L Aminian K, et al. Technical validation of real-world monitoring of gait: a multicentric observational study. BMJ Open 2021; 11: e050785. 10.1136/bmjopen-2021-050785 PMC8640671 34857567

[10] Goldsack JC Coravos A Bakker JP, et al. Verification, analytical validation, and clinical validation (V3): the foundation of determining fit-for-purpose for biometric monitoring technologies (BioMeTs). NPJ Digit Med 2020; 3: 55. 32337371 10.1038/s41746-020-0260-4 PMC7156507

[11] FDA. Patient focused drug development: collecting comprehensive and representative input. Rockville, MA: U.S. Department of Health and Human Services Food and Drug Administration, 2018.

[12] EMA. Qualification of novel methodologies for medicine development. Amsterdam: European Medicine Agency, 2022. Available from: https://www.ema.europa.eu/en/human-regulatory/research-development/scientific-advice-protocol-assistance/qualification-novel-methodologies-medicine-development-0.

[13] FDA. Clinical outcome assessment (COA): Frequently asked questions. Silver Spring, MA: Food and Drug Authority, 2022. Available from: https://www.fda.gov/about-fda/clinical-outcome-assessment-coa-frequently-asked-questions.

[14] WHO. Draft global strategy on digital health. Geneva: World Health Organisation, 2020.

[15] Keogh A Dorn JF Walsh L, et al. Comparing the usability and acceptability of wearable sensors among older Irish adults in a real-world context: observational study. JMIR Mhealth Uhealth 2020; 8: e15704. 32310149 10.2196/15704 PMC7199137

[16] Keogh A Argent R Anderson A, et al. Assessing the usability of wearable devices to measure gait and physical activity in chronic conditions: a systematic review. J Neuroeng Rehabil 2021; 18: 138. 34526053 10.1186/s12984-021-00931-2 PMC8444467

[17] Slevin P Kessie T Cullen J, et al. Exploring the potential benefits of digital health technology for the management of COPD: a qualitative study of patient perceptions. ERJ Open Res 2019; 5: 00239–2018. 31111039 10.1183/23120541.00239-2018 PMC6513035

[18] Slevin P Kessie T Cullen J, et al. A qualitative study of chronic obstructive pulmonary disease patient perceptions of the barriers and facilitators to adopting digital health technology. Digit Health 2019; 5: 2055207619871729. 31489206 10.1177/2055207619871729 PMC6710666

[19] Whitelaw S Pellegrini DM Mamas MA, et al. Barriers and facilitators of the uptake of digital health technology in cardiovascular care: a systematic scoping review. Eur Heart J Digit Health 2021; 2: 62–74. 34048508 10.1093/ehjdh/ztab005 PMC8139413

[20] Yadav L Haldar A Jasper U, et al. Utilising digital health technology to support patient-healthcare provider communication in fragility fracture recovery: systematic review and meta-analysis. Int J Environ Res Public Health 2019; 16: 4047. 31652597 10.3390/ijerph16204047 PMC6843966

[21] Gromisch ES Turner AP Haselkorn JK, et al. Mobile health (mHealth) usage, barriers, and technological considerations in persons with multiple sclerosis: a literature review. JAMIA Open 2021; 4: ooaa067. 34514349 10.1093/jamiaopen/ooaa067 PMC8423420

[22] Shorten A Smith J. Mixed methods research: expanding the evidence base. Evid Based Nurs 2017; 20: 74–75. 28615184 10.1136/eb-2017-102699

[23] Sim J Saunders B Waterfield J, et al. Can sample size in qualitative research be determined a priori? Int J Soc Res Methodol 2018; 21: 619–634.

[24] Boddy CR. Sample size for qualitative research. Qual Market Res Int J 2016; 19: 426–432.

[25] Robinson OC. Sampling in interview-based qualitative research: a theoretical and practical guide. Qual Res Psychol 2013; 11: 25–41.

[26] Knight J Baber C Schwirtz A, et al. The comfort assessment of wearable computers. 6th International Symposium on Wearable Computers 2002.

[27] Rabinovich RA Louvaris Z Raste Y, et al. Validity of physical activity monitors during daily life in patients with COPD. Eur Respir J 2013; 42: 1205–1215. 23397303 10.1183/09031936.00134312

[28] Nuske HJ Goodwin MS Kushleyeva Y, et al. Evaluating commercially available wireless cardiovascular monitors for measuring and transmitting real-time physiological responses in children with autism. Autism Res 2022; 15: 117–130. 34741438 10.1002/aur.2633 PMC9040058

[29] Ammann-Reiffer C Klay A Keller U. Virtual reality as a therapy tool for walking activities in pediatric neurorehabilitation: usability and user experience evaluation. JMIR Serious Games 2022; 10: e38509. 35834316 10.2196/38509 PMC9335180

[30] Mahs M Pithan JS Bergmann I, et al. Activity tracker-based intervention to increase physical activity in patients with type 2 diabetes and healthy individuals: study protocol for a randomized controlled trial. Trials 2022; 23: 617. 35907864 10.1186/s13063-022-06550-z PMC9338482

[31] Mercer K Giangregorio L Schneider E, et al. Acceptance of commercially available wearable activity trackers among adults aged over 50 and with chronic illness: a mixed-methods evaluation. JMIR Mhealth Uhealth 2016; 4: e7. 26818775 10.2196/mhealth.4225 PMC4749845

[32] Lee J Kim D Ryoo H-Y, et al. Sustainable wearables: wearable technology for enhancing the quality of human life. Sustainability 2016; 8: 466.

[33] Puri A Kim B Nguyen O, et al. User acceptance of Wrist-Worn activity trackers among community-dwelling older adults: mixed method study. JMIR Mhealth Uhealth 2017; 5: e173. 29141837 10.2196/mhealth.8211 PMC5707431

[34] Keogh A Sett N Donnelly S, et al. A thorough examination of morning activity patterns in adults with arthritis and healthy controls, using actigraphy data. Digit Biomark 2020; 4(3): 78–88. 33173843 10.1159/000509724 PMC7590760

[35] Davis F. Perceived usefulness, perceived ease of use and user acceptance of information technology. MIS Q 1989; 13: 319–340.

[36] Hennink M Kaiser BN. Sample sizes for saturation in qualitative research: a systematic review of empirical tests. Soc Sci Med 2022; 292: 114523. 34785096 10.1016/j.socscimed.2021.114523

[37] Bodine K Gemperle F. Effects of Functionality on Perceived Comfort of Wearables. Seventh IEEE International Symposium on Wearable Computers 2003.

[38] Rupp MA Michaelis JR McConnell DS, et al. The role of individual differences on perceptions of wearable fitness device trust, usability, and motivational impact. Appl Ergon 2018; 70: 77–87. 29866329 10.1016/j.apergo.2018.02.005

[39] Steinert A Haesner M Steinhagen-Thiessen E. Activity-tracking devices for older adults: comparison and preferences. Univers Access Inf Soc 2017; 17: 411–419.

[40] Schroedl CJ Yount SE Szmuilowicz E, et al. A qualitative study of unmet healthcare needs in chronic obstructive pulmonary disease. A potential role for specialist palliative care? Ann Am Thorac Soc 2014; 11: 1433–1438. 25302521 10.1513/AnnalsATS.201404-155BC PMC4298987

[41] Members of the MSitsCSG, Rieckmann P Centonze D, et al. Unmet needs, burden of treatment, and patient engagement in multiple sclerosis: a combined perspective from the MS in the 21st century steering group. Mult Scler Relat Disord 2018; 19: 153–160. 29241142 10.1016/j.msard.2017.11.013

[42] Harding R Selman L Beynon T, et al. Meeting the communication and information needs of chronic heart failure patients. J Pain Symptom Manage 2008; 36: 149–156. 18599259 10.1016/j.jpainsymman.2007.09.012

[43] Feehan LM Geldman J Sayre EC, et al. Accuracy of fitbit devices: systematic review and narrative syntheses of quantitative data. JMIR Mhealth Uhealth 2018; 6: e10527. 30093371 10.2196/10527 PMC6107736

[44] Nakagata T Murakami H Kawakami R, et al. Step-count outcomes of 13 different activity trackers: results from laboratory and free-living experiments. Gait Posture 2022; 98: 24–33. 36030707 10.1016/j.gaitpost.2022.08.004

