# Supplementary material for: Acceptability of wearable devices for measuring mobility remotely: Observations from the Mobilise-D technical validation study
Source: Digit Health. 2023 Feb 1;9:20552076221150745. doi: 10.1177/20552076221150745 (PMC9900162; doi:10.1177/20552076221150745)
Supplement: sj-docx-1-dhj-10.1177_20552076221150745 - Supplemental material for Acceptability of wearable devices for measuring mobility remotely: Observations from the Mobilise-D technical validation study [file sj-docx-1-dhj-10.1177_20552076221150745.docx]

Participant interviews

***Aim of the interview***

The aims of the semi-structured interview are to:

1. Ensure that the McRoberts Dynaport device is comfortable and acceptable to participants.
2. Explore the acceptability of wearable devices, for the purposes of healthcare monitoring, to participants in general.

Specifically for the Mobilise-D study, it is critical to understand whether the Dynaport device is acceptable for them to wear for a week. These questions are therefore being asked first. Depending on the time remaining in the interview, the general questions regarding the use of wearables as monitoring devices should be left until you have learned everything you wish to about the Dynaport device.

**Note:** Questions in this topic guide are included to answer the above aims. If the participant is open and talks freely, they may answer some of the questions without being asked. Therefore, depending on the person, not all of these questions need to be asked. If they begin to talk about topics that may be interesting or relevant to the above aims, please feel free to continue to explore these, even if there is no specific question linked to it. In contrast, if participants are not very open, some potential prompts have been included with the questions below. These prompts are there as an optional guide and do not need to be used.

***General points to remember regarding the interview process***

Questions for each aim have been listed accordingly:

- Main interview questions
  - These focus on the primary aim
  - They should form the basis of the interview
- Planned follow-up questions
  - These will help make the interview more specific if required
  - They may be useful to direct non-talkative participants

Spontaneous questions that result from the responses of participants obviously cannot be included in this guide, but are encouraged.

When completing the interviews please remember the following:

- Start with broad questions to generate discussion.
- Try to avoid questions with multiple options as this will lead them towards a specific answer
- If you need to confirm specific details, then you can use questions which require short responses.
- Try to find a quiet area to complete the interview if possible. If possible, please also try to be alone with the participant. However, if they request or need to have another person present (e.g. carer, spouse) then do not refuse them this.
- Bring a notebook or something to take notes as you go. This will help you to remember key phrases. Participants may also tell you stories while they complete the questionnaires, you may write down these words at the time and ask about them in the interview.
- Please be aware that participants may become upset during the interview. If this happens, let them continue if they can.
- When participants provide you with responses do not respond with subjective phrases such as ‘great’ or ‘that’s surprising’ as this may alter their future responses. Please only respond with unemotional responses such as ‘ok’ or ‘that’s interesting’.
- If participants remark that they are uncertain about something, please do not educate them during the interview. For example, if they remark that they do not know what the Dynaport was measuring, wait until the interview is complete before providing them with this information.

Interview guide

| Dynaport questions | |
| --- | --- |
| Aim: Ensure that the McRoberts Dynaport device is comfortable and acceptable to participants | |
| **Main questions** | |
| 1 | Can you describe your experience of using the Dynaport sensor in the last week? |
| 2 | Can you tell me what you liked about the device? Disliked?  *Prompts if needed*   - Size/weight - Attachment to body - Ease of use - Comfort |
| 3 | How did the device make you feel? / Can you describe what it felt like to wear the device?  *Prompts if needed*   - In social environments/at home - Interaction with daily activities - Emotions associated with being monitored |
| 4 | What you change about the device if you could? |
| **Follow-up questions**  **“**If you don’t mind, I’d like to ask you some specific details about the device.” | |
| 1 | How did you find the process of putting it on and taking it off? |
| 2 | How did the device influence your daily activities?  *Prompts if needed*   - How were they impacted? - How did this make them feel? |
| 3 | Can you tell me about any difficulties that you had with the device? |
| 4 | How did you feel about wearing the device for a week?  *Prompts if needed*   - How would they feel if it was longer? - Any concerns for the week? |
| **Closing question** | |
| 1 | Is there anything else you would like me to know about the Dynaport? |

| The use of wearable devices in healthcare questions | |
| --- | --- |
| Aim: Explore the acceptability of wearable devices, for the purposes of healthcare monitoring, to participants in general. | |
| **Main questions** | |
| 1 | Can you tell me about your experience of your health condition?  *Note:* Condition specific symptoms are listed at the end of this document. |
| 2 | Can you tell me about your experience of the care you’ve received for your condition?  *Prompts if needed*   - How do they feel about it? |
| 3 | Can you tell me about what sort of technology you currently use in your everyday life?  *Prompts if needed*   - How do you feel about using technology? - What would make you use technology more? Less? - Emotions associated with being monitored |
| 4 | What are your opinions on the use of technology in healthcare?  *Prompts if needed*   - What do you think it can be used for? |
| 5 | How would you feel about using technology to generate health information about yourself? (e.g. condition related smartphone app, self-reported outcomes platform, fitness tracker etc.),  *Prompts if needed*   - Why? - What would make you use it? - What would stop you from using it? - What would need to change for you to use it? |
| **Follow-up questions** | |
| 1 | How do you feel about capturing health information in your daily life, using a wearable remote monitoring device? |
| 2 | How do you think digital technology used in your daily life would influence how you manage your condition?  *Prompts if needed*   - How would it impact their relationship with their health care provider? - Integration into activities of daily living |
| 3 | How would you feel about sharing this data with your health care provider? What about researchers?  *Prompts if needed*   - Usefulness - Impact of this |
| **Closing question** | |
| 1 | Is there anything else you would like me to know about using technology in healthcare? |

Note for researchers

Some participants may mention or allude to symptoms that are common with their respective conditions. These may include, but are not limited to:

**Parkinson’s Disease**: risk of falls, episodes of ‘freezing gait’ where they feel like they cannot move forward, a tremor, reduced balance, slow walking speed, slowness of movement, muscular stiffness.

**PFF:** weakness, pain, reduced mobility

**CHD:** shortness of breath, fatigue, weakness, swelling in legs and ankles, irregular heartbeat, reduced ability to exercise, fluid retention, persistent cough.

**COPD**: shortness of breath, the need for home oxygen, wheezing, tightness in their chest, a chronic cough, increased mucus production, frequent infections, lack of energy, swelling, weight loss.

**MS:** muscle spasms or weakness, numbness and tingling, reduced mobility, fatigue, bladder and bowel issues, pain, depression.
